# Cardiac Magnetic Resonance Assessment of the Structural and Functional Cardiac Adaptations to Soccer Training in School-Aged Male Children

**DOI:** 10.1007/s00246-018-1844-5

**Published:** 2018-03-08

**Authors:** Marzena Barczuk-Falęcka, Łukasz A. Małek, Hubert Krysztofiak, Danuta Roik, Michał Brzewski

**Affiliations:** 1 Department of Pediatric Radiology, Medical University of Warsaw, Żwirki i Wigury 63A, 02-091 Warsaw, Poland; 2 Faculty of Rehabilitation, Józef Piłsudski University of Physical Education in Warsaw, Marymoncka 34, 00-968 Warsaw, Poland; 3 Department of Applied Physiology, Mossakowski Medical Research Centre PAS, Pawińskiego 5, Warsaw, Poland

**Keywords:** Children, Sport, Training, Athlete’s heart, Cardiac magnetic resonance, Adaptation

## Abstract

Physical training is associated with changes in cardiac morphology called the “athlete’s heart”, which has not been sufficiently studied in children. The aim of the study was to analyze cardiac adaptation to exercise in pre-adolescent soccer players. Thirty-six soccer players (mean age 10.1 ± 1.4 years) and 24 non-athlete male controls (10.4 ± 1.7 years) underwent cardiac magnetic resonance. Measurements of myocardial mass, end-diastolic and end-systolic volume, stroke volume and ejection fraction for left and right ventricle (LV, RV) were performed. Additionally, left and right atrial (LA, RA) areas and volumes were analysed. Relative wall thickness (RWT) was calculated to describe the pattern of cardiac remodeling. Interventricular wall thickness and LV mass were significantly higher in athletes, but remained within the reference (6.9 ± 0.8 vs. 6.2 ± 0.9 mm/√m^2^, *p* = 0.003 and 57.1 ± 7.4 vs. 50.0 ± 7.1 g/m^2^, *p* = 0.0006, respectively) with no changes in LV size and function between groups. The RWT tended to be higher among athletes (*p* = 0.09) indicating LV concentric remodeling geometry. Soccer players had significantly larger RV size (*p* < 0.04) with similar function and mass. Also, the LA volume (*p* = 0.01), LA area (*p* = 0.03) and LA diameter (*p* = 0.009) were significantly greater in players than in controls. Cardiac adaptations in pre-adolescent soccer players are characterized by an increased LV mass without any changes in LV size and systolic function, which is typical of resistance training with tendency to concentric remodeling. This is accompanied by increase of LA and RV size. It should be taken into account during annual pre-participation evaluation.

## Introduction

According to the World Health Organization (WHO), physical activity in school-aged children provides fundamental health benefits, improves cardiorespiratory and muscular fitness and reduces cardiovascular risk in the future life [1]. The WHO recommends at least 60 min of moderate- to vigorous-intensity physical activity per day, while activity greater than 60 min provides additional health benefits [1]. This may be in part explained by positive heart remodeling in relation to prolonged exercise. It has been shown that regular and extensive training is associated with changes in cardiac morphology called the “athlete’s heart”, which includes increased left (LV) and right ventricular (RV) cavity dimension, wall thickness and masses [2–4]. The degree of cardiac adaptation is related to the workload of training and usually appears already after several weeks of regular exercise. Depending on the type of sport being practiced, there are characteristic changes in the heart according to the Morganroth hypothesis from 1975 broadly adopted in the scientific and medical literature [4–6]. The original hypothesis differentiates eccentric left ventricular hypertrophy (LVH) observed in athletes performing endurance training from concentric LVH in athletes undergoing strength training [4]. In recent years several publications questioned this theory; it failed to support the cardiac phenotype expected in strength training. Some of them suggested that LV wall thickness is increased more in endurance than strength-trained athletes, others have reported no morphological changes in resistance-trained athletes [7–10]. This may be at least in part explained by the fact that some disciplines of sport (e.g., soccer, rugby, hockey) combine both endurance and strength training elements [4]. Therefore, there is an ongoing need to verify the Morganroth hypothesis with more accurate methods such as cardiac magnetic resonance (CMR) or 3-D echocardiography [3, 4, 6].

Unfortunately, almost all the studies have been carried only on adult and young adolescent players [2, 11]. Only single reports based on 2-D echocardiography analyse the impact of the short- and long-term training in children [12–15]. To our knowledge, the available literature does not provide any CMR (gold standard) data concerning the influence of long-term exercise on the heart in active school-aged children.

Soccer is the most popular sport in Europe [11], which requires combination of endurance, resistance and speed activity and differs from pure dynamic and static sports [4, 11, 13]. The effect of such combined exercise on cardiac remodeling has been less investigated. After the UEFA 2012 Euro championships that were organised in Poland and Ukraine, soccer became very popular among Polish children. Because of the significance of potential results, we have decided to address our research to a group of young soccer players.

The aim of our study is to investigate the effects of prolonged soccer training on cardiac morphology and function in school-aged players using CMR.

## Methods

Thirty-six male, Caucasian soccer players, age 8–12 years old (mean age 10.1 ± 1.4 years, mean BSA 1.18 ± 0.21 m^2^) volunteered to take part in the study. All of the participants have been engaged in regular training (2 × 90 min per week with 60 min league matches on weekends) for at least 2 years during most months of the year (except July and August). CMR examinations took place during their competitive (active) season.

The findings were compared with the 24 healthy male, Caucasian, age- and BSA-matched controls (mean aged 10.4 ± 1.7 years, mean BSA 1.23 ± 0.22 m^2^) who volunteered for the study. They have practiced recreational sport during school classes and seasonally, but were not engaged in any formal physical training or organized physical activity. All the subjects were healthy and were not taking any medications that would affect cardiovascular fitness. BSA was calculated according to the Mosteller formula [16].

CMR imaging was performed with a Siemens Magnetom Skyra 3T scanner (Siemens, Erlangen, Germany) including initial scout images, followed by cine steady-state free precession (SSFP) breathhold sequences in 2-, 3-, and 4-chamber views. The short axis was identified using the 2- and 4-chamber images and included the whole heart from ventricular apex up (including the atria). Imaging parameters were as follows: field of view 340 mm, matrix 208 x 256, repetition time approximately 39.24 ms, echo time 1.43 ms, flip angle 39 degrees, slice thickness 6 mm, gap 2 mm, in-plane image resolution 1.6 × 1.6, temporal resolution 25 phases per cardiac cycle. Quantitative measurements were preceded by venc-scout velocity range assessment in the aorta at the level of the sino-tubular junction [17].

Images were analysed with the use of dedicated software. Initially, short axis SSFP cine images were previewed from the base to the apex in a cinematic mode, then endocardial and epicardial contours for end-diastole and end-systole of both ventricles were manually traced. Trabeculae and papillary muscles were considered as ventricle cavities. Delineated contours were used for the quantification of LV and RV end-diastolic and end-systolic volumes (LVEDV, LVESV, RVEDV, RVESV), stroke volumes (LVSV, RVSV), ejection fractions (LVEF, RVEF) and masses (LVM, RVM). Obtained values were internally validated against flow measurement in the ascending aorta. Then the parameters were indexed for BSA and presented as LVEDVI, LVESVI, LVSVI, LVMI, and RVEDVI, RVESVI, RVSVI, RVMI.

The LV internal diameter (LVEDd), interventricular wall thickness (IVS) and LV posterior wall thicknesses (PWD) in end-diastole and aortic bulb (AB) diameter and ascending aorta (AA) diameter in end-systole were measured on the 3-chamber images and based on the above, the relative wall thickness (RWT) was calculated as [18, 19]:

$${\text{RWT}}=2 \times \rm {{PWD}/ {LVEDd.}}$$

In measuring IVS, care was taken to exclude right ventricular septal bands. In measuring the PWD, care was taken to exclude posterior wall chordae. A RWT was considered to be abnormal if > 0.42 [16, 17].

Subsequently, the volumes of the left and right atria (LA, RA) were also manually traced on end-systole and end-diastole to get the maximal and minimal volumes (LAVol_max, LAVol_min, RAVol_max, RAVol_min). Additionally the area of both atrias was measured on end-systole in 4-chamber view (LA_area, RA_area) and LA diameter was measured on end-systole in 3-chamber view (LAD). Area and volume measurements (two- or three-dimensional) were adjusted to BSA and linear measurements (one-dimensional) to the square root of BSA and were presented as LAVol_max_I, LAVol_min_I, RAVol_max_I, RAVol_min_I and LA_area_I, RA_area_I and LADI.

Furthermore, to simply assess RV volume and systolic function, two parameters were measured: (1) RV diameter in the 4-chamber view in end-diastole in the widest possible place below tricuspid annular ring and indexed as RVDI, and (2) tricuspid annular plane systolic excursion (TAPSE) towards the apex in the 4-chamber view, measured by marking position of RV free wall and tricuspid annulus junction point in end-diastole and in end-systole and measurement of the distance between the two positions. The following criteria served as reference ranges [20].

All of the CMR measurements were assessed by a European Society of Cardiology certified Level 3 expert with 9 years of expertise in the field (Ł.A.M.) and by a second reader (M.B-F.) with over 1 year of expertise.

Nominal variables were presented as absolute numbers and percentages, continuous variables in the form of means and standard deviations (SD, in the case of variables with normal distribution) or medians and interquartile range (IQR, in the case of variables with non-normal distribution). Normality of distribution was assessed with means of the Kolmogorov–Smirnoff test. Comparison of percentage distribution of individual nominal variables between groups was made with the *χ*^2^ test or the Fisher exact test, depending on the abundance of the individual subgroups. In order to assess differences between means and medians, the Student’s *t* test for independent variables or the Mann–Whitney test for independent variables was used, respectively. In order to assess the correlation between continuous variables, the r-Pearson or Spearman test were applied, respectively. Inter-observer and intra-observer variability for CMR measurements of LVMI, RVEDVI and LA volume was assessed respectively in ten and five randomly selected patients using the Bland–Altman repeatability analysis method and interclass correlation coefficient (ICC). Bias was expressed as an average ± 2 SD. All tests were bilateral and *p* < 0.05 was considered as statistically significant. MedCalc software version 10.0.2.0 (MedCalc, Mariakerke, Belgium) was used for statistical analyses.

Sample size was determined with G*power (version 3.1.3; Dusseldorf, Germany). We estimated that a sample size of at least 31 individuals in the study group and 17 controls would have a power of 90% to detect an approximately 10% between-group difference (10 ml with SD ± 10 ml for LVEDV or RVEDV, 5 g with SD ± 5 g for LVM, 2.5 g with SD ± 2.5 g for RVM and 3 cm^3^ with SD ± 3 cm^3^ for LA or RA volume) given two-tailed *α* = 0.05 and 1.75 allocation between groups.

## Results

The CMR studies of the 36 male soccer players were analyzed. There were no significant differences in age and BSA between athletes and controls (Table 1). Left and right ventricular morphologic and functional parameters of both groups are summarized in Table 1.

End-diastolic LV volume was increased in two athletes (5.6%). All soccer players had normal LV systolic function. There were no significant differences in LV size and LVEF between study and control group.

**Table 1:** Left and right ventricular morphologic and functional parameters of study and control groups

Parameter	Study group *n* = 36	Control group *n* = 24	*p*
Age, years ± SD	10.1 ± 1.4	10.4 ± 1.7	0.46
BSA ± SD	1.18 ± 0.21	1.23 ± 0.22	0.35
LVEDVI, ml/m^2^ ± SD	80.7 ± 10.9	78.9 ± 11.1	0.53
LVESVI, ml/m^2^ ± SD	29.7 ± 6.2	28.2 ± 6.5	0.37
LVSVI, ml/m^2^ ± SD	51.0 ± 9.0	50.4 ± 7.8	0.80
LVMI, g/m^2^ ± SD	57.1 ± 7.4	50.0 ± 7.1	0.0006
LVEF, % ± SD	63.9 ± 4.5	64.4 ± 5.4	0.69
RVEDVI, ml/m^2^ ± SD	90.9 ± 13.4	84.2 ± 10.5	0.036
RVESVI, ml/m^2^ ± SD	39.2 ± 7.9	34.7 ± 7.8	0.037
RVSVI, ml/m^2^ ± SD	51.5 ± 8.5	49.5 ± 6.3	0.31
RVMI, g/m^2^ ± SD	18.6 ± 3.5	20.8 ± 10.9	0.38
RVEF, % ± SD	56.6 ± 5.1	59.0 ± 6.0	0.12
RVDI, mm/√m^2^ ± SD	32.7 ± 4.0	32.1 ± 2.6	0.51
TAPSE, mm/√m^2^ ± SD	21.3 ± 3.9	20.8 ± 3.8	0.64
LVEDd, mm/√m^2^ ± SD	38.3 ± 3.3	37.2 ± 3.2	0.21
IVSd, mm/√m^2^ ± SD	6.9 ± 0.8	6.2 ± 0.9	0.003
PWDd, mm/√m^2^ ± SD	7.1 ± 1.3	6.5 ± 1.3	0.08
RWT ± SD	0.35 ± 0.1	0.32 ± 0.1	0.09
LAVol_max_I, ml/m^2^ ± SD	34.2 ± 8.3	30.6 ± 6.7	0.11
LAVol_min_I, ml/m^2^ ± SD	15.1 ± 5.4	11.8 ± 3.6	0.01
RAVol_max_I, ml/m^2^ ± SD	38.3 ± 10.9	36.1 ± 9.7	0.48
RAVol_min_I, ml/m^2^ ± SD	20.0 ± 5.0	17.1 ± 5.1	0.06
LA_area_I, cm^2^/m^2^ ± SD	12.5 ± 2.6	11.1 ± 2.0	0.03
RA_area_I, cm^2^/m^2^ ± SD	11.7 ± 2.7	11.1 ± 1.9	0.31
LADI, mm/√m^2^ ± SD	25.4 ± 2.9	22.7 ± 4.0	0.009
AB, mm/√m^2^ ± SD	22.3 ± 2.5	22.5 ± 2.2	0.78
AA, mm/√m^2^ ± SD	19.9 ± 2.0	20.2 ± 2.6	0.59
MPA, mm/√m^2^ ± SD	19.0 ± 2.5	19.6 ± 1.7	0.29
LPA, mm/√m^2^ ± SD	12.2 ± 1.3	11.8 ± 1.5	0.40
RPA, mm/√m^2^ ± SD	12.5 ± 1.7	12.3 ± 1.4	0.70

*AA* ascending aorta, *AB* aortic bulb, *BSA* body surface area, *IVS* interventricular septum, *LA* left atrium, *LADI* 3-chamber left atrial diameter index, *LPA* left pulmonary artery, *LVEDVI* left ventricular end-diastolic volume index, *LVEF* left ventricular ejection fraction, *LVEDd* left ventricular end-diastolic diameter, *LVESVI* left ventricular end-systolic volume index, *LVMI* left ventricular mass index, *LVSVI* left ventricular stroke volume index, *MPA* main pulmonary artery, *PWD* posterior wall diameter, *RA* right atrium, *RPA* right pulmonary artery, *RWT* relative wall thickness, *RVDI* right ventricular diameter index, *RVEF* right ventricular ejection fraction, *RVEDVI* right ventricular end-diastolic volume index, *RVESVI* right ventricular end-systolic volume index, *RVMI* right ventricular mass index, *RVSVI* right ventricular stroke volume index, *SD* standard deviation, *TAPSE* tricuspid annular plane systolic excursion

End-diastolic RV volume was increased in four soccer players (11.1%). All soccer players had normal RV systolic function. However, when compared to controls, the study group had significantly higher RV size (*p* = 0.036 for RVEDVI and *p* = 0.037 for RVESVI).

LVMI in end-diastole was significantly increased (*p* = 0.0006) in players as compared to controls (57.1 ± 7.4 vs. 50.0 ± 7.1 g/m^2^). However all LVMI values in athletes were within the reference range (38–86 g/m^2^) according to normal values published by Kawel-Boehm et al. [20]. Similar results were obtained when we compared LV myocardial thickness measured as IVS and PWD. The IVS was thicker and PWD tended to be thicker in athletes than in the non-training subjects (*p* = 0.003 for IVS, *p* = 0.08 for PWD).

**Fig. 1:**
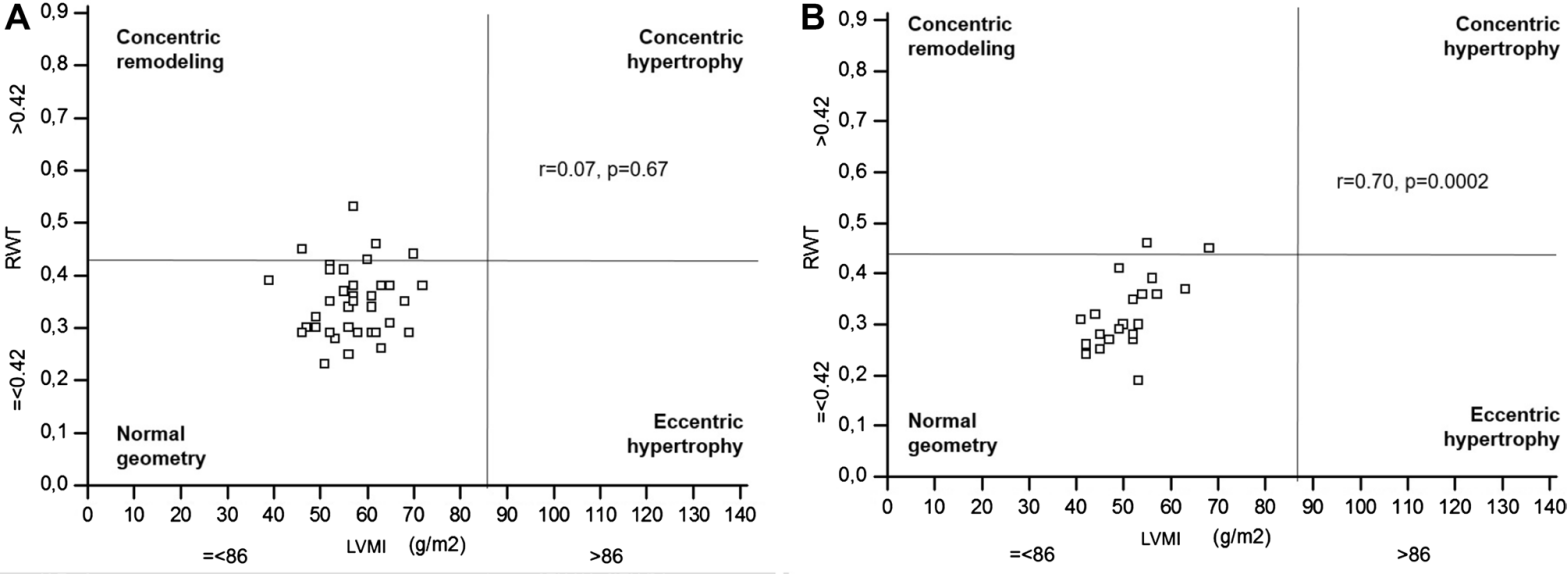
Left ventricular geometry according to relative wall thickness and left ventricular mass in the study group and in the control group. **a** Study group, **b** Control group. *RWT* relative wall thickness, *LVMI* left ventricular mass index

Also, the RWT tended to be higher among athletes (*p* = 0.09) and in five of them (13.9%) exceeded 0.42, indicating LV concentric remodeling geometry (Fig. 1).

In the study group there was no correlation between RWT and LVMI, in contrast to the control group where good correlation of these parameters was observed (*r* = 0.7, *p* = 0.0002). There were no significant differences in RVMI between both groups.

The values of minimal LA volume (*p* = 0.01), LA area (*p* = 0.03) and LADI measured in 3-chamber view (*p* = 0.009) were significantly higher in soccer players than in controls. There was also a tendency to higher RA minimal volume among athletes (*p* = 0.06).

There was a very good agreement for LVMI between two readers and for the repeated assessment of the same operator (Ł.A.M.). The Bland–Altman plots showed average difference in estimates of − 0.5 ± 2.8% for inter-observer variability and 0.0 ± 1.8% for intra-observer variability. ICC was 0.92 for inter-observer variability and 0.96 for intra-observer variability. Similar results were obtained for other analyzed parameters (RVEDVI and LA volume).

## Discussion

Morphological cardiac changes in response to physical conditioning depend on the type, duration and intensity of training [21]. In 1975 Morganroth et al. described LV adaptation and concluded that pure endurance training in adults is associated with eccentric hypertrophy, whereas pure resistance training is characterized by concentric hypertrophy [4, 5]. The different types of the myocardial remodeling of popular sport disciplines such as soccer, combine features of endurance and strength training and are less well investigated with CMR, so the current observations should be interpreted in the context of the relatively limited literature, especially in children.

This is the first study to examine cardiac effects of soccer on the pre-adolescent population using CMR. We showed that the LV wall thicknesses (IVS, PWD) were significantly increased in soccer players compared to controls with no changes in LV cavity size and function, which, in correlation with increased LVMI (by about 14%), suggest cardiac changes characteristic for resistance sports.

Our results are different from the findings of Zdravkovic et al. who used echocardiography to analyse 94 pre-adolescent male footballers (mean age 12.85 ± 0.84 years). The author showed significant increase of absolute LV dimensions (*p* < 0.001), whereas there were no differences in absolute values of LV septal and posterior wall thicknesses and LVM [2]. Our results are more in line with those of an echocardiographic study published by Krustrup et al., who analysed cardiac adaptation in children 9–10 years old in response to a 10-weeks period of soccer training [12]. The only changes found included increased PWD and IVS, without any differences in LV diameter and function. Also, Sharma et al. noted one of the greatest increases of LV wall thickness in soccer players among different type of athletes [22].

Our findings suggest that soccer, at least in the early years of practice, is a type of sport predominantly related to resistance remodeling. This is in contrast with the traditional view published in Task Force 8: Classification of Sports by Mitchell et al. showing soccer as a low-static and high-dynamic type of sport therefore predisposing more to endurance remodeling according to the Morganroth hypothesis [23].

Nevertheless, none of our study subjects had LVM above the reference range signifying concentric hypertrophy. According to the guidelines of the American Society of Echocardiography, LV geometry in athletes with normal LVM can be classified into two groups based on the RWT values: normal cardiac geometry (RWT ≤ 0.42) or concentric remodeling (RWT > 0.42) [24]. In our study most soccer players had normal LV geometry, however concentric remodeling was observed in 13.8% of subjects.

Similar results were recently demonstrated by Finocchiaro et al., who used echocardiography to analyze athletes practicing approximately 40 sport disciplines subdivided into static, dynamic, or mixed (8% of them were soccer players classified as a dynamic type of sport). The study demonstrated that most athletes had normal LV geometry, although concentric remodeling or hypertrophy in male athletes engaged in dynamic sports was also observed (15% of subjects) [18].

We should also note that the cardiac remodeling with predominant LVM increase may result from specificity of training in the pre-adolescent soccer players, which differs significantly from training plans in adults. It consists mainly of ball control exercises, jumps and run-ups with lots of repetitions and systematicity to improve motion automation with less attention to fitness training and long-distance running. A CMR study by Sharf et al. on adult soccer players showing remodeling typical for endurance and resistance training may support this hypothesis [11].

Understanding RV adaptation to intense training has been, similar to the LV, previously documented in adult athletes [25–27], but there are only single reports in children based on echocardiographic study [12, 14].

We observed significant increase of RV size. Our findings are in line with the findings of D’Ascenzi et al. [14] but different from the ones published by Krustrup et al., where no changes in RV diameter were observed [12]. While the first was based on practicing competitive swimmers at the regional level, the later one included only a short training intervention (10 weeks), which could be insufficient to demonstrate relevant changes. Our study adds up to increasing evidence that RV may be more prone to remodeling than LV and not only in life-time endurance athletes as shown by LaGerche et al., but also at the very beginning of the sporting life [25]. Finally, it is also possible that increase of the RV size may be the first marker of eccentric remodeling preceding LV size increase observed in adults soccer players as demonstrated earlier [11].

The LA and RA have received even less attention that RV. Iskandar et al. in their meta-analysis have observed either LA diameter or LA volume increase in elite adult athletes [28]. The largest average LA diameters were reported in endurance athletes, but LA size was also increased in strength-trained and combined trained athletes [28]. Similar results were found in children by Zdravkovic et al., who observed increased LA diameter in pre-adolescent soccer players [2] and in the work of D’Ascenzi et al. [15] who demonstrated a biatrial increase in juvenile swimmers. This is in agreement with our results, where all—linear, two-dimensional and volumetric measures of the LA size were significantly increased and accompanied by a trend in the RA size increase.

There are several limitations of the present study. First of all, our study group size was relatively smaller in comparison to above quoted publications based on echocardiography [2, 12, 21]. However, as demonstrated by calculations above, our study has sufficient power to demonstrate significant differences between groups. It is also notable that CMR is considered to be substantially more precise in terms of cardiac morphological assessment than echocardiography. This is also related to low inter- and intra-observer variability, as demonstrated in our study.

Secondly, there are limited normal values available for CMR variables in children, especially for measures of myocardial mass, and these cannot be readily extrapolated from adult data. We have based our study on the most commonly used ones [20].

Finally, our study was based on a male Caucasian population. Previous research demonstrated that there are gender and race differences in cardiac remodeling in relation to exercise. Therefore, our results should not be extended to the general population.

## Perspectives

In summary, the present study is the first CMR study investigating the pattern of juvenile myocardial remodeling in school-aged male soccer players. Cardiac adaptations in that group are characterized by an increased LV wall thickness and mass without any changes in LV size and systolic function, which is typical for resistance training with tendency to concentric remodeling. This is accompanied by increase of LA and RV size, which completes the picture of a juvenile athlete’s heart. These findings should be taken into account during pre-participation evaluation to lower the risk of false positive findings, where physiological adaptation may be misinterpreted as a sign of the disease with all the consequences. Finally, it would be also interesting to see how these early changes transfer to later life and health benefits, which warrant further studies.
